# Revealing the dominant long noncoding RNAs responding to the infection with *Colletotrichum gloeosporioides* in *Hevea brasiliensis*

**DOI:** 10.1186/s13062-019-0235-z

**Published:** 2019-04-15

**Authors:** Hongyan Yin, Xiaodong Zhang, Bei Zhang, Hongli Luo, Chaozu He

**Affiliations:** Hainan Key Laboratory for Sustainable Utilization of Tropical Bioresources, Institute of Tropical Agriculture and Forestry, Hainan University, Haikou, 570228 China

**Keywords:** *Hevea brasiliensis*, Co-expressed network, Long noncoding RNAs, microRNAs

## Abstract

**Background:**

Rubber tree (*Hevea brasiliensis*) acts as an important tropic economic crop and rubber tree anthracnose, mainly caused by *Colletotrichum gloeosporioides,* is one of the most common fungal disease, which leads to serious loss of rubber production. Therefore, the investigation on disease resistance is of great worldwide significance. In the past decades, substantial progress has been made on coding gene families related with plant disease resistance. However, in rubber tree, whether the disease resistance mechanism involves noncoding RNAs, especially long noncoding RNAs (lncRNAs), still remains poorly understood.

**Results:**

Here, we modeled the development of *H. brasiliensis* leaf samples inoculated with *C. gloeosporioides* at divergent stages, explored to identify the expressed ncRNAs by RNA-seq, and investigated the dominant lncRNAs responding to the infection, through constructing a co-expressed network systematically. On the dominant lncRNAs, we explored the potential functional role of lncRNA11254 recruiting the transcription factor, and that lncRNA11041 and lncRNA11205 probably stimulate the accumulation of corresponding disease responsive miRNAs, and further modulate the expressions of target genes, accompanying with experimental examination.

**Conclusions:**

Take together, computational analyses in silico and experimental evidences in our research collectively revealed the responsive roles of dominant lncRNAs to the pathogen. The results will provide new perspectives to unveil the plant disease resistance mechanisms, and will presumably provide a new theoretical basis and candidate prognostic markers for the optimization and innovation of genetic breeding for rubber tree*.*

**Reviewers:**

This article was reviewed by Ryan McGinty and Roland Huber.

**Electronic supplementary material:**

The online version of this article (10.1186/s13062-019-0235-z) contains supplementary material, which is available to authorized users.

## Background

Rubber tree (*Hevea brasiliensis*) as one of important tropic economic crops is the only source for ever-growing demand for natural rubber, which is an important industrial and commercial raw material [30, 46]. It is documented that diseases could cause more than 25% loss of rubber yields [56]. Rubber tree anthracnose, mainly caused by *Colletotrichum gloeosporioide,* is the most common fungal disease and leads to serious loss of rubber production [8]. During the evolution, it is believed that plants have evolved to possess exquisite resistance mechanisms in the biotic stress, and transcriptome profiling is efficient to reveal the responses to pathogens [41, 43]. In the past decades, great progress has been continuously made on the metabolic pathways and specific coding genes including jasmonic acid (JA), salicylic acid (SA), mitogen-activated protein kinase (MAPK) and so on [1, 35], whereas the whole molecular response mechanism, especially in rubber tree, still remains poorly understood.

These years, accompanying with the next-generation sequencing technology, emerging evidences have accumulated showing that ncRNAs, which were previously regarded as “dark matter” [20], tend to play roles in numerous biological processes [10, 32] among divergent species ranging from prokaryotes [5], fungi [18, 50], animals [2], and plants [31, 54]. In general, the definition of ncRNAs is based on sequence length, which divides ncRNAs into long noncoding RNAs (lncRNAs) that have more than 200 nucleotides (nt) and the remaining small RNAs, including microRNAs (miRNAs), small nucleolar RNAs (snoRNAs) and piwiRNAs (piRNAs). In plants, miRNAs are usually documented to participate in responding to stresses at transcriptional and posttranscriptional levels [3, 9, 19]. These years, lncRNAs are continually reported to be responsive to biotic or abiotic stresses [10, 14, 51]. Especially, a recent research has discovered that a lncRNA *ELENA1* directly influence the plant immunity associated with elf18, a pathogen-associated factor that induces defense responses in *Arabidopsis* [33]. These studies indicate that in-depth investigation on putative stress responsive ncRNAs, especially the lncRNAs, is helpful for better understanding the disease resistance mechanism. However, whether the molecular response mechanism of *H. brasiliensis* involves lncRNAs, still remains unclear.

Here, in our study, we modeled the development of *H. brasiliensis* (Reyan7–33-97) leaf samples being inoculated with *C. gloeosporioides* at different stages, accomplished the assembly of their transcriptomes, and explored to identify the expressed ncRNAs strictly in *H. brasiliensis* leaves. Furthermore, we systematically explored the dominant lncRNAs responding to the pathogen through constructing a co-expressed network. Additionally, on the dominant lncRNAs, we investigated the potential functional role of lncRNA11254 with the transcription factor (TF), and examined the possibility that lncRNA11041 and lncRNA11205 stimulate the accumulation of corresponding disease responsive miRNAs, and further modulate the expressions of target genes, accompanying with experimental verifications.

## Methods

### Plant materials, inoculation, cDNA library construction and sequencing

The inoculated samples were adopted in the same condition at 4 continuous stages (initial inoculation, 1 day after inoculation, 3 days after inoculation, and 5 days after inoculation) of the immature leaves from *Hevea* trees (Reyan7–33-97 planted at the experimental plantation of Hainan University, Danzhou, Hainan, China). Before inoculation, an appropriate amount of mycelium of *C. gloeosporioides* was cultured in PD liquid media for 2 days and the conidia were collected by centrifugation at 4000 rpm. The spore solution was adjusted to concentration at 5 × 10^5^ CFU/mL with sterile water and was sprayed evenly on the rubber tree leaves. The inoculated samples grew at 25 °C and in no lower than 90% huminity. According to the set time, the samples were collected and were kept in liquid nitrogen immediately for RNA extraction. The total RNA was extracted using method of LiCl precipitation [24]. The cDNA libraries were constructed according to the manufacturer’s protocols. Sequencing was conducted via Illumina HiSeq 2000 platform. Meanwhile, 5 uninfected leaf samples of Reyan7–33-97 as control group were from SRA (https://www.ncbi.nlm.nih.gov/sra) (SRA IDs: SRR3136159, SRR3136185, SRR3136188, SRR3136190, SRR3136192).

### Transcriptome profiling and identification of up-regulated responsive ncRNAs

After obtaining raw paired-end reads, quality control was conducted by both fastqc (http://www.bioinformatics.babraham.ac.uk/projects/fastqc/) and NGS QC Toolkit v2.3.3[38]. All sequencing reads maintained were aligned to the rubber tree genome released in 2016 [46] with Tophat2 (v2.1.1) [23]. Cufflinks [49] and Stringtie [40] were used in turn to assemble the transcripts and merge the transcripts among different samples multiple times. The expression levels were measured using FPKM (fragments per kilobase of transcript per million mapped reads). Ballgown was used to examine the responsive expressed coding genes (RGs), along with responsive expressed noncoding RNAs (RncRNAs) [17, 39], with FPKM > 0.1 and FC > 2 (fold changes between our inoculated sample series and control ones) (Additional file 1: Table S1).

To identify the ncRNAs, three steps were conducted as follows: 1) Longest transcripts of individual genes or potential RNAs were selected and annotated by NR database with BLASTX; 2) Longest transcripts were scanned against InterPro’s signatures, provided by multiple different databases and applications, along with assignments of GO (Gene Ontology) and KEGG (Kyoto Encyclopedia of Genes and Genomes) [16]; 3) Any given query with an above hit in last two steps was excluded and coding potentials of the remained sequences were calculated by CPC (Coding Potential Calculator) [25].

### Phylogenetic analysis, pathway assignment, RNA-protein pair predication, motif detection and miRNA binding sites scan

Considering the close evolutionary relationship between *R. communis* and *H. brasiliensis* (both belong to *Euphorbiaceae*) (Fang, et al. 2016) and their well-sequenced genomes (from RefSeq; https://www.ncbi.nlm.nih.gov/refseq), we used the homologs of these two species to calculate the nonsynonymous substitution rate (Ka) and synonymous substitution rate (Ks) by KaKs_Calculator using the model of MA [58]. The homologs were detected by BLASTN (Overlap> 0.7, E < 1e-3, Identity> 0.8). Pathway assignments were conducted by KAAS (http://www.genome.jp/kegg/kaas) using the BBH (bi-directional best hit) method. RNA-protein pairs were predicted by catRAPID [4]. The motifs matched to the lncRNA were detected by MAST (http://meme-suite.org/tools/mast). In rubber tree, the miRNAs were previously identified [29], and all the miRNAs were listed in Additional file 1: Table S2. Potential binding sites were conducted by an online toolkit psRNATarget (http://plantgrn.noble.org/psRNATarget). Details of tools and databases we utilized in this study were summarized into Additional file 1: Table S3.

### Real-time qRT-PCR, relative expression calculation

In inoculated samples and non-inoculated control leaves at divergent stages, transcriptional levels of lncRNAs and representative genes were measured by real-time quantitative reverse transcription PCR (qRT-PCR) at divergent stages, respectively. Three independent biological replicates for each condition were carried out, for two times. First-strand cDNA was synthesized from 2 μg DNase-treated total RNA (Thermal reversed first-strand cDNA synthesis Kit) and 50 ng cDNA was taken as template for PCR. Relative expression (RE) was calculated using the 2^−ΔΔC^_T_ method.

### Construction of mesophyll protoplast system

Mesophyll protoplast system refers to the transient mesophyll protoplast expression system. The transient mesophyll protoplast system could be applied to analyze gene function of rubber tree, a perennial plant for which the applicable transformation system was limited. In our study, we used our improved method to isolate high quality rubber tree mesophyll protoplasts [57]. The transient expression of exogenous DNA constructs in this mesophyll protoplast system was observed by using green fluorescent protein, and was detected by immunoblot.

## Results and discussion

### Identification and characterization of expressed ncRNAs in infected rubber tree leaves

To investigate the response dynamics of rubber tree to pathogens, we generated transcriptome-sequencing datasets of *H. brasiliensis* leaves infected with *C. gloeosporioides* (Methods). In transcriptome profiling, the corresponding mapping ratio to the genome of individual sample was 90.1, 90.3, 90.1, 90.4% (Table 1) and the assembled transcriptome yielded a total of 35,441 uniquely expressed sequences, involving 81,569 transcripts. Among the uniquely expressed sequences, 32,272 sequences were annotated with more than one hit against NR database and 22,996 sequences with at least one hit by InterPro. 32,347 sequences were assigned to a given functional annotation by either NR or InterPro. Ultimately, for 3107 ncRNA candidates retained, after calculation of corresponding coding potentials, 3094 sequences were identified as expressed ncRNAs in rubber tree leaves.

**Table 1:** Preliminary results in transcriptome profiling and noncoding identification

Name		Number
Mapping ratios	0D	90.1%
	1D	90.4%
	3D	90.3%
	5D	90.4%
Number of transcripts		81,569
Number of Coding Genes and Noncodings RNAs		35,441
Coding Genes (Genes)		32,347
Hit on NR		32,272
Hit by InterPro		22,996
Noncodings (Noncoding RNAs)		3094

**Fig. 1:**
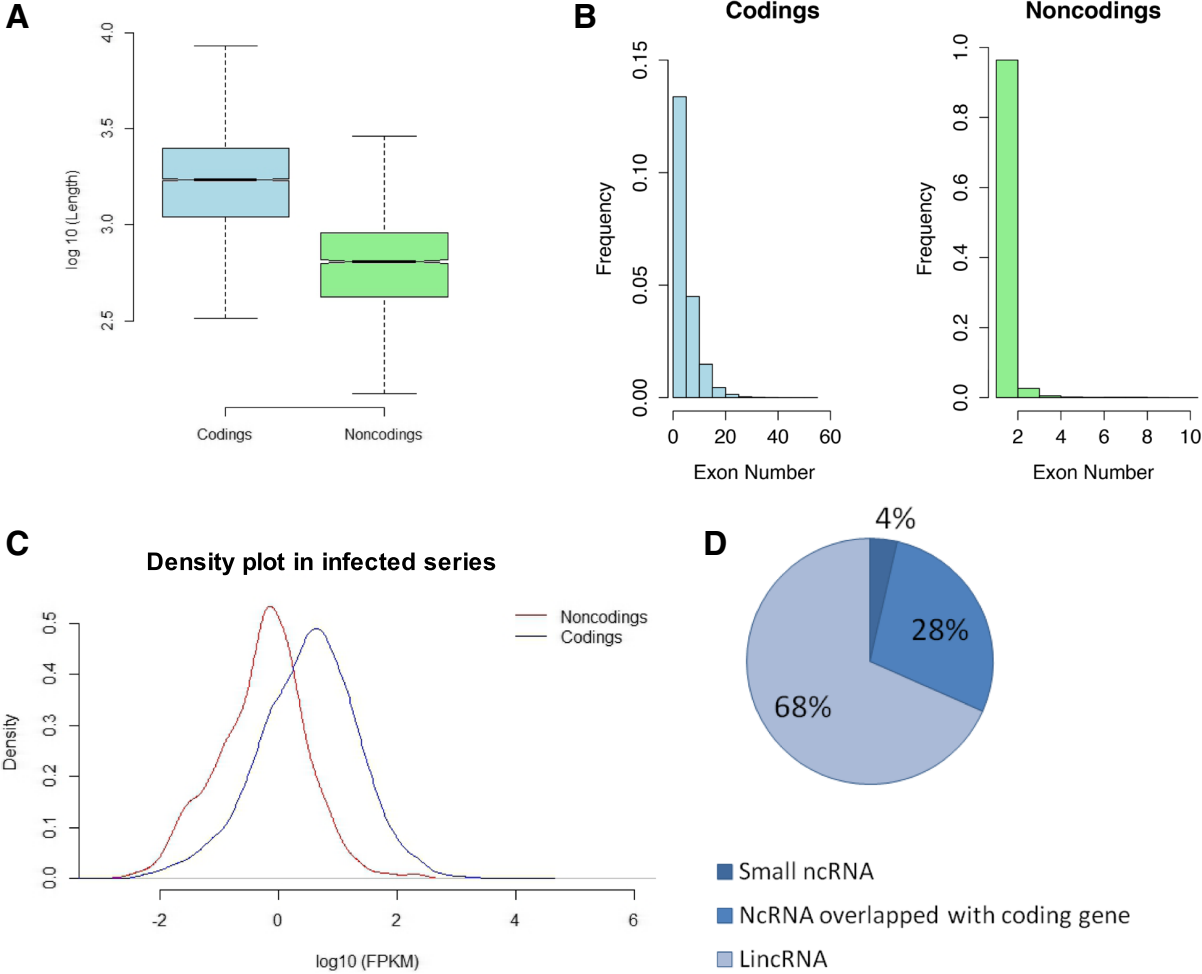
Characterization of expressed transcripts from coding genes and noncoding RNAs (ncRNAs) in infected samples. **a** Length distribution for transcripts of coding genes and ncRNAs in boxplots. **b** Exon number distribution for transcripts of coding genes and ncRNAs in histograms. **c** Density plot for average expressions of coding transcripts and ncRNAs in our infected series samples. **d** Classification of identified expressed ncRNAs

Evidences accumulate that the ncRNAs are in correspondence with genome complexity [7]. Therefore, a better characterization on their sequence components is of great significance. We assumed that the sequence components of coding transcripts would exhibit differently with ncRNAs. Coincided with our assumption, firstly, on the length distribution for transcripts of coding genes and ncRNAs, we found the average transcript length of expressed ncRNAs was ~ 500 bp, whereas that of coding genes was 1.7 Kb, obviously longer than ncRNAs (Fig. 1a). Moreover, on the exon number distributions, majority of ncRNAs tended to contain less introns, and a small proportion of ncRNAs were possessed of multiple exons (Fig. 1b). On the GC content, average GC content of coding transcripts was 41.54%, relatively higher than that of ncRNAs, 37.77%.

In addition, it has been reported that the expression levels of ncRNAs are usually 30-folds to 60-folds lower than those of mRNA in *Arabidopsis thaliana* [51]*.* Consistently, in rubber tree leaves inoculated with *C. gloeosporioides*, it showed that ncRNAs expressed at relatively low levels, in comparison with coding genes in infected leaves (Fig. 1c) and control (Additional file 2: Figure S1). Besides, among the ncRNAs, about 4% of 3094 expressed ncRNAs were small ncRNAs ranging from 85 nt to 197 nt; 68% were lncRNAs located at the intergenic regions (lincRNAs); 28% were lncRNAs overlapped with coding genes at sense strand or antisense strand (Fig. 1d).

### Exploration of dominant responsive up-regulated ncRNAs

**Fig. 2:**
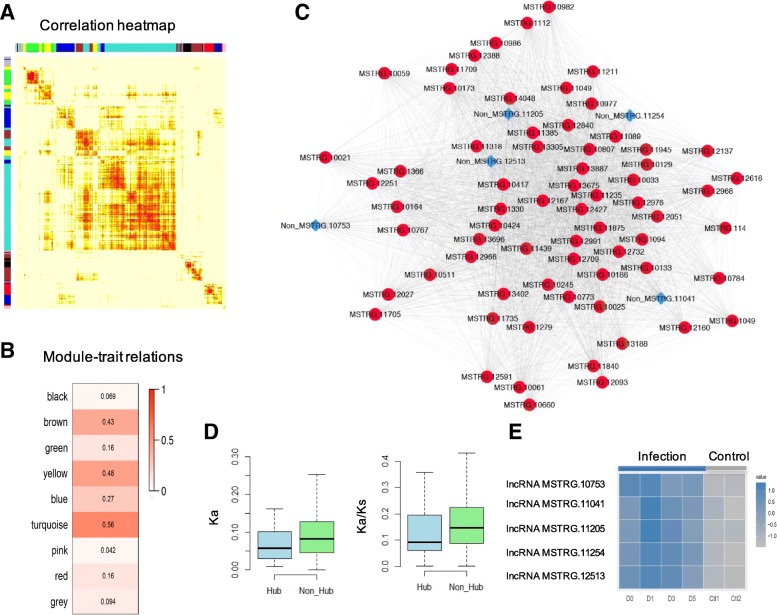
Exploration of dominant responsive ncRNAs. **a** Modules clustered in the co-expressed network shown by different colors in the heatmap. **b** Correlation between individual module and the infection. **c** Hub responsive expressed coding genes (RGs) and responsive expressed ncRNAs (RncRNAs) shown in module “turquoise”. Top 5% nodes, ranking by the number of significant related nodes, were selected and defined as hub RncRNAs or RGs. RGs were represented by red dots and RncRNAs were represented by blue diamond dots. **d** Comparison of the nonsynonymous substitution rate (Ka) and ratio of Ka to synonymous substitution rate (Ks) between hub and non hub RGs. **e** Expression distributions of dominant lncRNAs in the heatmap

Capturing the related expression patterns of genes under different conditions are effective for revealing functionally related ones [45, 55]. In transcriptome profiling (Methods), 3445 up regulated uniquely disease responsive expressed sequences were obtained, including 3154 RGs and 291 RncRNAs. Accordingly, based on multiple gene expression profiles, we deciphered the underlying correlation relationships between individual RncRNAs and RGs using Pearson’s correlation coefficient. The resulting correlations were generated to a weighted correlation network, with each node representing a RncRNA or RG, and the highly connected ones were assigned into modules by the soft-threshold method [28]. The co-expressed network was confirmed to possess a reliable scale-free property with evaluated S (G) > 0.7. Consequently, the network was resulted into 9 modules (represented by different colors in the heatmap; Fig. 2a). Nodes in module “turquoise” were found to be related with the responses to the pathogen mostly (*R* > 0.58; *P* < 0.05; Fig. 2b) through the trait-based significance measurement [28]. In module “turquoise”, top 5% nodes possessing highly significantly related partners were selected and defined as hub ones, indicating their dominances. Finally, we obtained 67 hub RGs and 5 hub RncRNAs (Fig. 2c), revealing the dominant responsive roles of ncRNAs to the pathogen, associated with coding genes.

On the identified hub RGs, most of them were assigned to the functions involving response mechanisms (Additional file 1: Table S4), such as MAPK signaling pathway, inferring the remarkable roles of detected hub RGs. Furthermore, compared with non-hub RGs, we found that hub RGs explored lower nonsynonymous substitution rate and stronger purifying selection pressure (Methods; Fig. 2d). Collectively, the detected remarkable roles related to response mechanisms and the stronger purifying selection proved the dominance of hub RGs. Additionally, on the hub RncRNAs, interestingly, we found that all of these five RncRNAs were lncRNAs (length > 200 nt) and their expression levels were clearly showed to be up-regulated in infected samples (Fig. 2e).

### Potential function of dominant RncRNAs recruiting the TF in trans

**Fig. 3:**
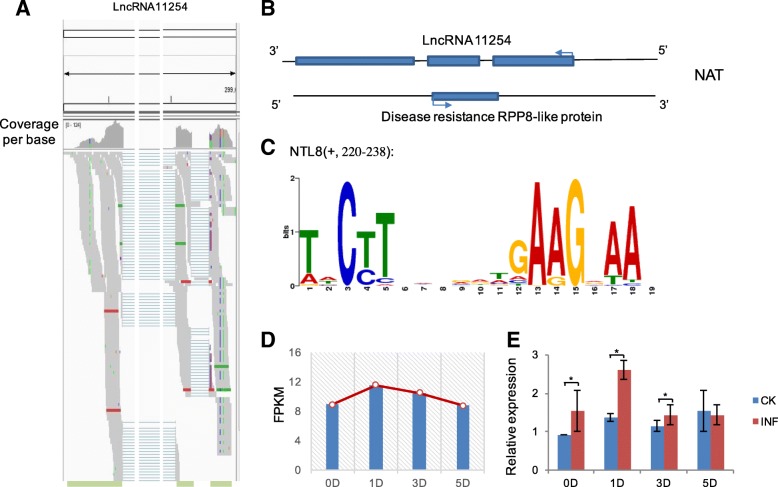
Potential function of lncRNA11254 associated with transcription factor responding to the pathogen. **a** Overview of aligned reads for lncRNA11254 using Integrative Genomics Viewer (IGV) with coverage of per base in sample 1D being shown. Other hub lncRNAs were shown in Additional file 3: Figure S2. **b** The structure of scaffold10290 coving lncRNA11254 and the protein coding gene on the antisense strand. **c** Motif detection on lncRNA11254. **d** FPKM values of lncRNA11254 along the infection at different stages. **e** The relative expression by real-time quantitative reverse transcription PCR (qRT-PCR) in non-inoculated control (CK) and infected samples (INF). Values were presented as the mean ± SE of three independent experiments and * indicated significant differences (*P* < 0.05)

Large scale of comparative genome sequences of multiple species enabled functional inferences [15]. However, owing to the low conservation of lncRNAs among species [52], it is inefficient to explore the functional roles from the perspective of phylogeny. Considering that the locus of a lncRNA is helpful to infer its regulatory activities [11, 26], we exanimated their genomic loci of these hub lncRNAs. Firstly, the overviews of aligned reads mapped to the hub lncRNAs using Integrative Genomics Viewer (IGV) [42] showed the high performance of mappings (Fig. 3a and Additional file 3: Figure S2). Among the these five dominant responsive lncRNAs, we found that lncRNA MSTRG.11254 (lncRNA11254) possessed multi-exons, located at the antisense strand on scaffold0290 and overlapped with an annotated ORF of RPP8-like protein (Fig. 3b), tending to be a natural antisense transcript (NAT), whose transcript was from the opposite strand of a protein-coding gene [11, 53].

To identify whether the lncRNA play functional roles in *cis* or in *trans* [26], we estimated the potential interactions between these hub lncRNAs and proteins nearby (Methods). The result showed that none lncRNAs possessed interaction partners with nearby proteins. However, among theses five lncRNAs, a motif from NTL8, a membrane-associated NAC transcription factor from *Arabidopsis thaliana*, could matched to lncRNA11254 significantly (*P* < 0.05, *Q* < 0.01; Fig. 3e). Furthermore, according to the interaction predication, lncRNA11254 was found to interact with NTL8 protein (Z-score = 0.77). Considering that the locus of NTL8 was not on the same scaffold with lncRNA11254, the motif detection indicated that lncRNA11254 probably played a responsive role in *trans* to the infection. Recently, NTL8 has been reported to act as a novel regulator in stress response [12, 37] and regulate MYB family (TRY and TCL1) in *Arabidopsis* [48]. However, the orthologs of TRY and TCL1 were not detected in rubber tree, resulting that the regulations in rubber tree still need further examination. Additionally, our transcriptome profiling revealed that lncRNA11254 was up-regulated during the infection and was especially highly expressed at D1 stage (Fig. 3c). Consistently, the result of RE using qRT-PCR showed that lncRNA11254 was also up-regulated at stages 0D, 1D, 3D, especially at 1D stage (Fig. 3d) in inoculated leaves. To ensure the result, we independently resampled the inoculated leaves with three replicates, re-conducted the qRT-PCR and got the similar expression pattern. The result showed that, although the relative expression levels differed, the expression patterns keep consistent (Additional file 4: Figure S3A). Collectively, our exploration in silico combined locus examination with functional predication possibly provided an access to reveal the function of lncRNA11254. We deciphered the potential function in *trans* of the dominant NAT lncRNA11254, recruiting the TF, responding to the infection in rubber tree and this result is coincided with a previous report that NATs participated in respond to biotic or abiotic stresses [10].

### Functional inference of dominant RncRNAs associated with miRNAs

**Fig. 4:**
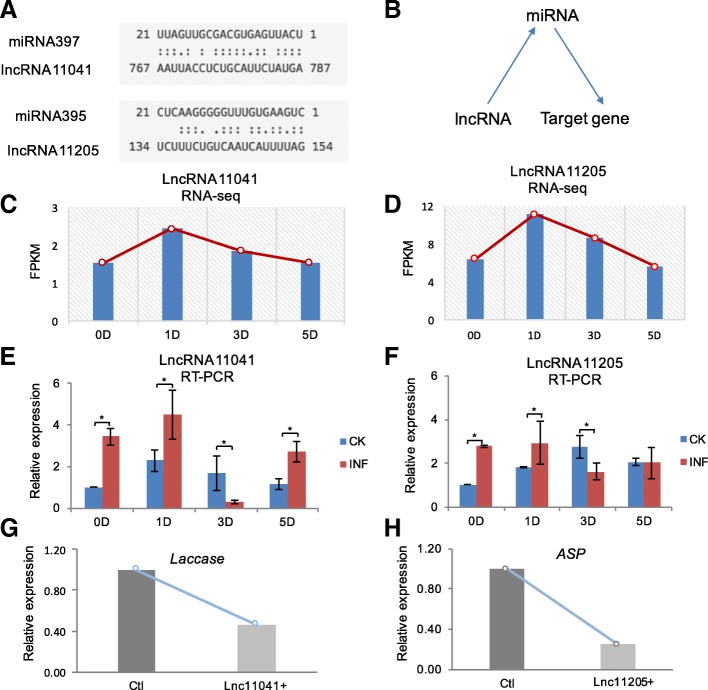
Functional inference of dominant RncRNAs associated with miRNAs. **a** Potential miRNA binding sites identification for lncRNA11041 and lncRNA11205. **b** Model of functional roles of lncRNA and miRNAs, associated with target genes. **c** FPKM values of lncRNA11041 along the infection at different stages. **d** FPKM values of lncRNA11205 along the infection at different stages. **e** The relative expressions (REs) of lncRNA11041 by real-time quantitative reverse transcription PCR (qRT-PCR) in non-inoculated control (CK) and infected samples (INF). **f** The REs of lncRNA11205 by qRT-PCR in CK and INF. **g** The REs of *Laccase* in rubber tree mesophyll protoplasts overexpressing lncRNA11041 (lnc11041+) and control (ctl). **h** The REs of *ASP* in rubber tree mesophyll protoplasts overexpressing lncRNA11205 (lnc11205+) and control (ctl). All the used miRNAs of rubber tree were listed in Additional file 1: Table S2. Values were presented as the mean ± SE of three independent experiments and * indicated significant differences (*P* < 0.05)

In addition to the possible functional roles through binding to proteins, these years, accumulating studies have revealed that lncRNAs might have functions associated with miRNAs [7, 10]. Therefore, we tended to explore the potential miRNA binding sites on these five dominant responsive lncRNAs (Methods). The result (Fig. 4a) showed that lncRNA MSTRG.11205 (lncRNA11205) and lncRNA MSTRG.11041 (lncRNA11041) possessed binding sites to miR395 and miR397, respectively. Remarkably, on these two miRNAs, miR397 is reported to be up-regulated in response to the biotic stresses [13, 27] and *Laccase* is documented to be its target gene in higher plants [34]. MiR395 and its target gene, *APS,* are usually reported to response to the sulfate-deprived conditions [21, 44]. Accordingly, we hypothesized that dominant responsive lncRNAs, lncRNA11041 and lncRNA11205, involved in the responses to the pathogen associated with miRNAs (Fig. 4b), and further regulated the expressions of the downstream coding genes.

Transcriptome profiling revealed that lncRNA11041 and lncRNA11205 were up-regulated during the infection (Fig. 4c-d). By qRT-PCR, compared with the non-inoculated control, lncRNA11041 and lncRNA11205 were up-regulated and tended to possess similar expression patterns with the result from transcriptome profiling (Fig. 4e-f, Additional file 4: Figure S3B-S3C). Additionally, we found that the response time differed at 3D stage between lncRNA1104/lncRNA11205 and lncRNA11254, possibly due to their different functional roles in the complicated immune defense system during the multistage hemibiotrophic infection from the pathogens [6, 22, 36]. To further investigate their potential functional roles on corresponding miRNAs, we detected the expression of *Laccase*, the reported target gene of miR397, in rubber tree mesophyll protoplasts overexpressing lncRNA11041(lnc11041+). Intriguingly, the result showed that the *Laccase* was significantly down-regulated (Fig. 4g). Similarly, the *ASP* was also found to be down-regulated in lncRNA11205-overexpressed protoplasts (lnc11205+; Fig. 4h). Consistently, the relative expression of miR397 and miR395 showed to be obviously elevated in corresponding rubber tree mesophyll protoplasts (Additional file 5: Figure S4). Taken together, these results inferred the possibility that lncRNA11041 and lncRNA11205 stimulated the quick accumulation of corresponding responsive miRNAs, which further modulate the expressions of their target genes during the response to the pathogen.

Generally, in response to abiotic and biotic stress, the involvement of miRNAs has been previously reported, regulating their target genes [13, 47]*.* Notably, in our study, the reported disease associated miRNAs in tropical plant leaves [13, 21, 27, 44] were also found to play significant roles in response to the pathogen in rubber tree, associated with the lncRNAs. Our functional predications of lncRNAs associating with miRNAs and the experimental investigations probably provide new insights into the response mechanism of rubber tree and facilitate deeper understanding on the dynamic roles of lncRNA in the complex plant disease resistance.

## Conclusions

Facilitated by high-throughput sequencing technology, tremendous progresses have been made in the function of plant ncRNAs. Here, based on the analyses in silico and experiment examinations, we performed comprehensive investigation on dominant lncRNAs responding to the pathogen and explored their functional roles. Despite this, functions of dominant lncRNAs were still not fully uncovered, especially for the lncRNA MSTRG.12513 and lncRNA MSTRG.10753. To better understand the complex disease resistance dynamics, more efforts are still in need including more experimental approaches, in-depth investigations, and continued explorations in the transgenic rubber tree with disease resistance abilities. Overall, deciphering lncRNAs responding to the pathogen is of great significance to unveil the molecular mechanism of disease resistance, presumably providing a new theoretical basis and candidate prognostic markers for the optimization and innovation of genetic breeding for rubber tree*.*

## Reviewers’ comments

### Reviewer’s report 1

Ryan McGinty

Reviewer comments:

*Major recommendations: The authors compare RNA-seq data generated from their infected leaf samples to RNA-seq data from a mock control group downloaded from the NCBI Short Read Archive (SRA). This represents the greatest weakness in the study. The RNA extraction methods used by control study (which were found only after following several links) appear to be different than those used in this study. Additionally, the control study provides details on handling of the leaf tissues prior to RNA extraction, and this information is absent in the present study. This is especially concerning, because RNA tends to be quickly degraded, and different species of RNA may be more or less resistant to degradation. Several features of RNA that are central to their analysis of the data are also factors that affect degradation, including the length of the RNA, whether it is highly-structured, whether it is capped and polyadenylated, whether it is packaged for transport,* etc. *It therefore seems likely that a number of important variables differ between the control study and the present study, and the resulting RNA-seq data may not be directly comparable. I would recommend that the authors repeat their experiments and generate their own control data under precisely the same conditions as their treatments. It would be advisable to harvest all plant samples together, mock-treat the controls alongside their treatments and extract RNA at the same time. It is also important to show that the quality of the extracted RNA is similar between all samples. Additionally, it appears that each treatment condition represents a single replicate. Replicates are explicitly mentioned in the qRT-PCR section, so it must be assumed that they were not performed for the RNA-seq experiment. This represents another considerable source of uncertainty.*

**Authors’ response:** *Thank you for the comment. Firstly, in our study, the inoculated samples were adopted in the same condition at 4 continuous stages (0D to 5D) of the immature leaves from Hevea trees (Reyan7–33-97 planted at the experimental plantation of Hainan University, Danzhou, Hainan, China). Considering that the mock control samples without inoculation in exactly same condition were not used in RNA-seq, the inoculated samples at 4 continuous stages were cross-referenced. Moreover, given that the inoculation lasting 5 days (0D to 5D) covers different leaf development stages, we used the data from SRA database (Reyan7–33-97 planted at the experimental plantation in Danzhou, Hainan, China; covering different leaf development stages) to reduce the negative effect from the leaf development. By computational analyses, using RNA-seq data from public database together with our data not only enabled us to identify the candidates of long coding RNAs, but also helped us to examine the dominance among the co-expression relationships. Just as you pointed out, in the control study, prior to leaf harvest, the trees had been tapped every three days for two years, whereas our leaves were harvested from rubber tree without being tapped. For experimental verifications were used to exclude false positives from computational data analyses as much as possible, we think the tapping did not affect the reliability on lncRNAs identification and their responsive roles, even if it would affect the expression of lncRNAs. Indeed, the consistent qRT-PCR results in the inoculated leaves confirmed the expression patterns of the lncRNAs we identified in computational analyses. In this study, to ensure the data reliability from a single replicated RNA-seq, we explicitly mentioned the replicates of qRT-PCR in this section. Here again, to ensure the robustness of the result, we independently resampled the inoculated leaves, with three more replicates and re-conducted the qRT-PCR verification. The result showed that, although the relative expression levels differed, the expression patterns of the lncRNAs (lncRNA11254, lncRNA11041, lncRNA11205) were similar with our original qRT-PCR results. Collectively, we think our result is reliable. The qRT-PCR results on the resampled inoculated leaves were summarized into Additional file* *4* *: Figure S3.*

*It would also be advisable to report more details on the experimental methods used. For example, there is no information provided on the inoculation with C. gloeosporioides, simply that it was inoculated.*

**Authors’ response:** *Thanks for the suggestion. An appropriate amount of mycelium of C. gloeosporioides was cultured in PD liquid media for 2 days and the conidia were collected by centrifugation at 4000 rpm. The spore solution was adjusted to concentration at 5 × 10*^*5*^ *CFU/mL with sterile water and was sprayed evenly on the rubber tree leaves. The inoculated samples grew at 25 °C and in no lower than 90% huminity. According to the set time, the samples were collected and were kept in liquid nitrogen immediately for RNA extraction. We have added the details in the manuscript on page 6.*

*The method of RNA extraction cites a paper from 1963 which may be the original reference for using LiCl to extract RNA, but is not useful as a current protocol for RNA extraction from plant material.*

**Authors’ response:** *Thanks for the comment. We have updated the reference.*

*And, as mentioned above, it is unclear how the plant material was grown, acquired and/or handled prior to RNA extraction. The experiments are not repeatable from this level of detail.*

**Authors’ response:** *Thanks for pointing it out. The samples were adopted in the same condition at 4 continuous stages (0D to 5D) of the immature leaves from Hevea trees (Reyan7–33-97 planted at the experimental plantation of Hainan University, Danzhou, Hainan, China). The inoculation with C. gloeosporioides was carried out in an inoculation room by the sprayer. The inoculated samples grew at 25 °C and in no lower than 90% huminity. According to the set time (initial inoculation, 1 day after inoculation, 3 days after inoculation, and 5 days after inoculation), the samples were collected and were kept in liquid nitrogen immediately for RNA extraction. We have added the details in the manuscript on* ***page 6*** *.*

*Minor recommendations: In several areas of the text, the experimental logic is not explained. Why was the genome of R. communis chosen for comparison the genome of H. brasiliensis?*

**Authors’ response:** *Thanks for the comment. In the phylogenetic analyses, gene pairs, which are usually homologs between close species, are used in examining the selection pressure* [58]*. In our analyses, we used the homologs between R. communis and f H. brasiliensis, for their close evolutionary relationship (both belong to Euphorbiaceae) as documented (Fang, et al. 2016). We have provided more details on* ***pages 7 and 8****.*

*Figure legend 4 mentions a “Mesophyll protoplast system” which is not mentioned anywhere in the Results and Discussion section. What is it and why was it used?*

**Authors’ response:** *Thanks for the comment. Mesophyll protoplast system refers to the transient mesophyll protoplast expression system. It has become a powerful tool for rapid gene functional analysis, and it has been used successfully in several plant species. Moreover, the transient mesophyll protoplast system could be applied to analyze gene function of rubber tree, a perennial plant for which the applicable transformation system was limited. In our study, we used our improved method to isolate high quality rubber tree mesophyll protoplasts* [57]*. The transient expression of exogenous DNA constructs in this mesophyll protoplast system was observed by using green fluorescent protein, and was detected by immunoblot. We have provided more details about Mesophyll protoplast system on* ***pages 8 and 9****.*

*In addition, the text names a number of tools and databases without explaining their content or purpose, or why they were used in this context. The manuscript as written requires a lot of prerequisite knowledge, and should be changed to be more easily understood by a broader audience.*

**Authors’ response:** *Thanks for the suggestion. To provide more prerequisite knowledge, we have summarized the descriptions of tools and databases we utilized in the study into Additional file* *1* *: Table S3. And we also have polished the manuscript to make it more easily understood.*

*Minor issues: There are numerous grammatical issues in the text.*

**Authors’ response:** *Thanks for the comment. We also have polished the manuscript.*

### Reviewer’s report 2

Roland Huber

Reviewer comments: *The authors propose 2 types of potential mechanism of action for the identified lncRNAs, one being a natural antisense transcript (NAT) lncRNA to the RPP8 gene, and two lncRNAs interacting with miRNA. The authors show that all 3 lncRNAs exhibit elevated relative expression levels in response to infection. Interestingly, relative expression levels for the lncRNA11041 and lncRNA11205 appear to be elevated initially and drop at the 3 day point, whereas lncRNA11254 (RPP8 antisense) appears consistently elevated for the first 3 days. I think it would be interesting for the authors to comment on possible reasons for this.*

**Authors’ response:** *Thanks for the comment. Colletotrichum employs multistage hemibiotrophic infection strategy to the plants* [36]*: the fungus initially grows biotrophically inside living epidermal cells after melanized appressoria breach the host cuticle and cell wall and finally it enters a destructive necrotrophic phase in which host tissues are destroyed by cell-wall-degrading enzymes. During the process, plants have also established a complicated immune defense system during co-evolution with pathogens, including PAMP-triggered immunity (PTI) and effector-triggered immunity (ETI)* [6]*, involving numerous metabolic pathways. It is believed that plants have developed branch points and interactions in the metabolic pathways controlling the biosynthesis and accumulation of related hormones and antimicrobial compounds so that a sustained defense response can be maintained* [22]*. In our computational analyses, lncRNA11254 probably participate in the response mechanisms through interaction with proteins, whereas, lncRNA11041 and lncRNA11205 play their roles, associated with miRNAs. The difference on 3 days after inoculation of these lncRNAs possibly results from their different functional roles during the above complicated host–microbe interactions. We have provided this discussion in the manuscript on* ***page 14.***

*The authors postulate that the presence of lncRNA11205 and lncRNA11041 may lead to faster accumulation of responsive miRNAs (miRNA397 and miRNA395 respectively), as they observe down regulation of target genes of these miRNAs. However, they do not try to directly prove such accumulation. I think additional efforts to demonstrate that this is indeed the mechanism of action would greatly strengthen the paper.*

**Authors’ response:** *Thanks for the suggestions. By qRT-PCR, we respectively detected the expression of miR397 in rubber tree mesophyll protoplasts overexpressing lncRNA11041 (lnc11041+), and the expression of miR395 in rubber tree mesophyll protoplasts overexpressing lncRNA11205 (lnc11205+), with the comparison of control (ctl). Coincide with our assumption, the result showed that miRNAs were obviously elevated when the corresponding lncRNAs were over expressed. We have provided the descriptions in the manuscript on page 14 and the results were summarized into Additional file* *5* *: Figure S4.*

*Minor issues: While the manuscript is readily comprehensible, it contains some grammatical issues throughout the text that the authors should correct.*

**Authors’ response:** *Thanks for the comment. We have polished the manuscript.*

## Additional files

Additional file 1: **Table S1.** Sample information for our datasets and datasets from public databases. **Table S2.** 115 miRNA of rubber tree used in our study. **Table S3.** Descriptions of the tools and databases used in the study. **Table S4.** Functional annotations for identified hub responsive coding genes. (XLS 58 kb)

Additional file 2: **Figure S1.** Density plot for average expressions of coding transcripts and ncRNAs in control group samples (Methods). (JPG 9 kb)

Additional file 3: **Figure S2.** Overviews of aligned reads for lncRNA MSTRG.10753, lncRNA MSTRG.12513, lncRNA MSTRG.11041 and lncRNA MSTRG.11205 using Integrative Genomics Viewer (IGV) with coverage of per base in Sample 1D being shown. (JPG 653 kb)

Additional file 4: **Figure S3.** The relative expressions by real-time quantitative reverse transcription PCR (qRT-PCR) in re-sampled non-inoculated control (CK) and inoculated samples (INF) of (A) lncRNA11254, (B) lncRNA11041 and (C) lncRNA11205. Values were presented as the mean ± SE of three independent experiments and * indicated significant differences. (*P* < 0.05) (PDF 15 kb)

Additional file 5: **Figure S4.** The relative expressions of miRNAs in rubber tree mesophyll protoplasts. (A) The relative expressions of miRNA397 in rubber tree mesophyll protoplasts overexpressing lncRNA11041 (lnc11041+) and control (ctl). (B) The relative expressions of miRNA395 in rubber tree mesophyll protoplasts overexpressing lncRNA11205 (lnc11205+) and control. (PDF 12 kb)

## Abbreviations

FPKM: Fragments per kilobase of transcript per million mapped reads
lncRNA: Long noncoding RNA
NAT: Natural antisense transcript
ncRNA: Noncoding RNA
qRT-PCR: Real-time quantitative reverse transcription PCR
RE: Relative expression
RG: Responsive expressed coding gene
RncRNA: Responsive expressed noncoding RNAs
TF: Transcription factor
