# Supplementary figures and images for: Revealing the dominant long noncoding RNAs responding to the infection with Colletotrichum gloeosporioides in Hevea brasiliensis
Source: Biol Direct. 2019 Apr 15;14:7. doi: 10.1186/s13062-019-0235-z (PMC6466799; doi:10.1186/s13062-019-0235-z)

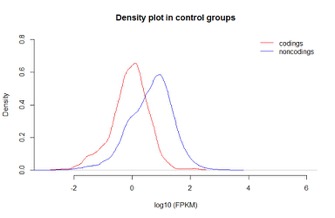

Supplement: Supplementary file 2 — Figure S1. Density plot for average expressions of coding transcripts and ncRNAs in control group samples (Methods). (JPG 9 kb) [file 13062_2019_235_MOESM2_ESM.jpg]

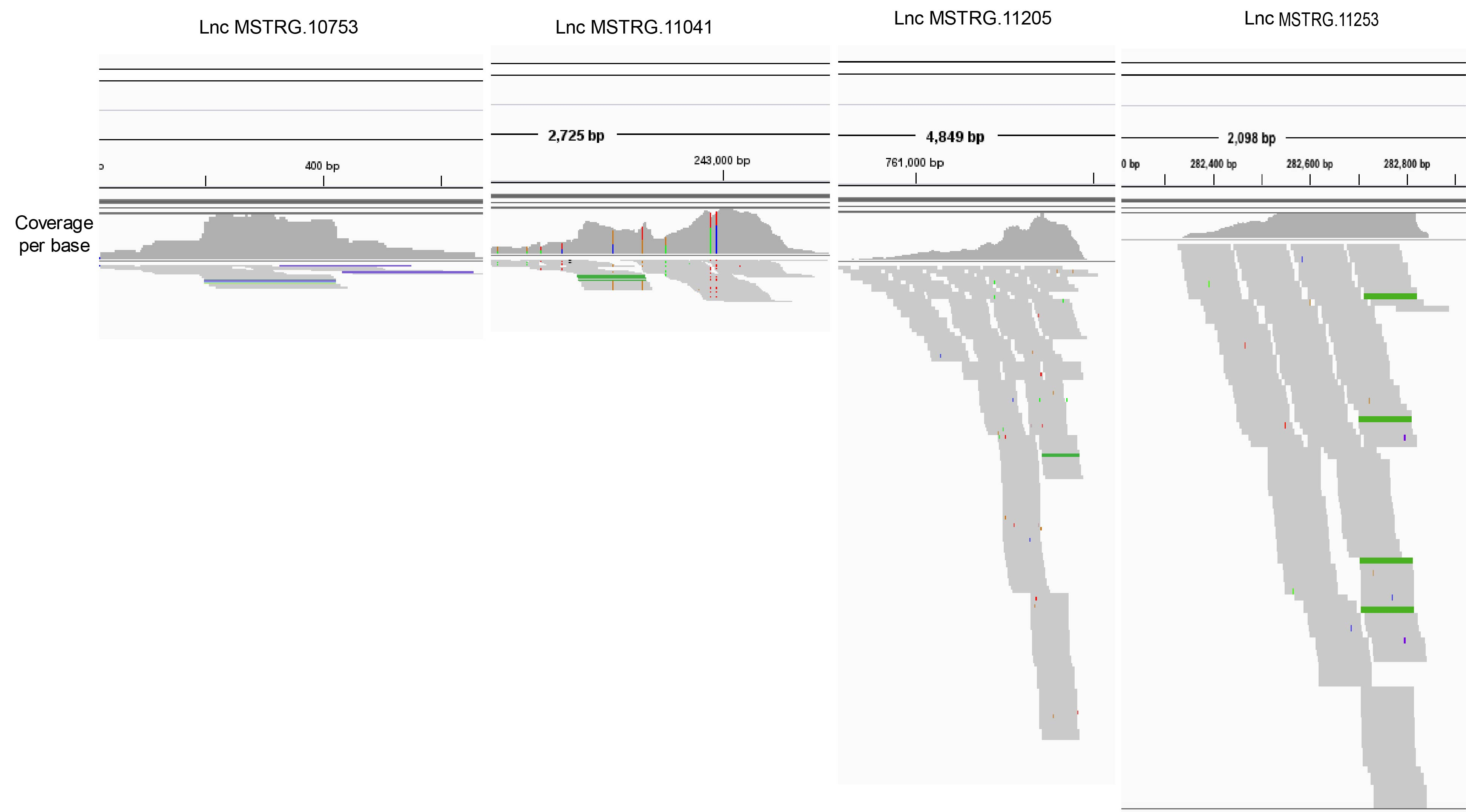

Supplement: Supplementary file 3 — Figure S2. Overviews of aligned reads for lncRNA MSTRG.10753, lncRNA MSTRG.12513, lncRNA MSTRG.11041 and lncRNA MSTRG.11205 using Integrative Genomics Viewer (IGV) with coverage of per base in Sample 1D being shown. (JPG 653 kb) [file 13062_2019_235_MOESM3_ESM.jpg]

**A** LncRNA11254  
RT-PCR

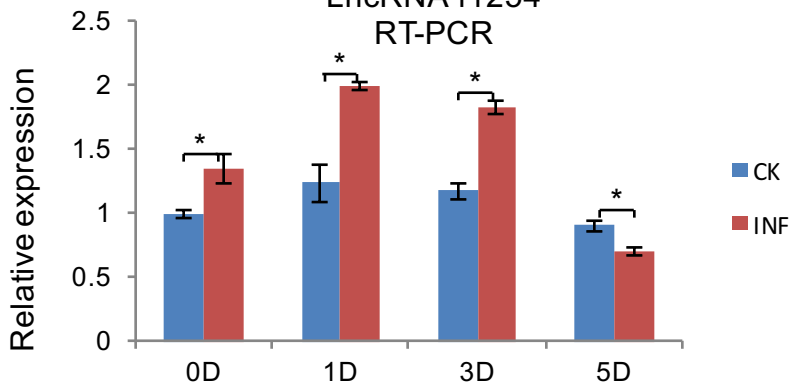

**B** LncRNA11041  
RT-PCR

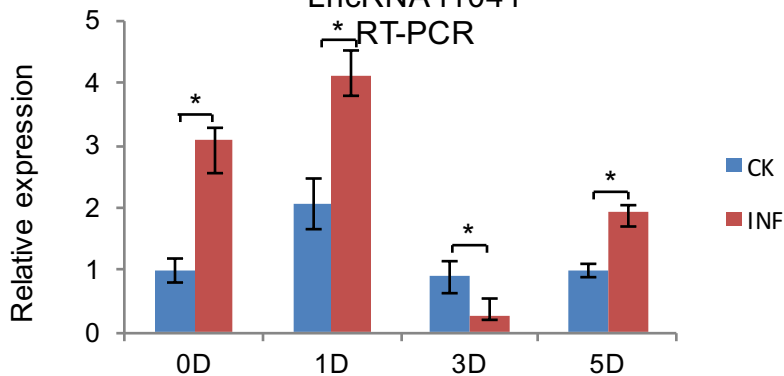

**C** LncRNA11205  
RT-PCR

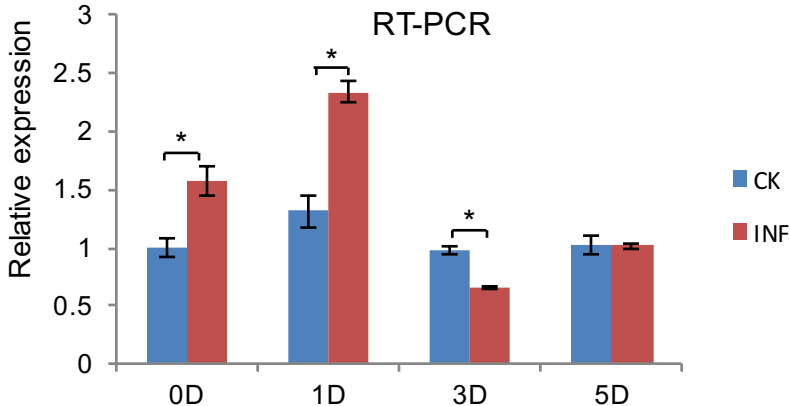

Supplement: Supplementary file 4 — Figure S3. The relative expressions by real-time quantitative reverse transcription PCR (qRT-PCR) in re-sampled non-inoculated control (CK) and inoculated samples (INF) of (A) lncRNA11254, (B) lncRNA11041 and (C) lncRNA11205. Values were presented as the mean ± SE of three independent experiments and * indicated significant differences. (P < 0.05) (PDF 15 kb) [file 13062_2019_235_MOESM4_ESM.pdf]

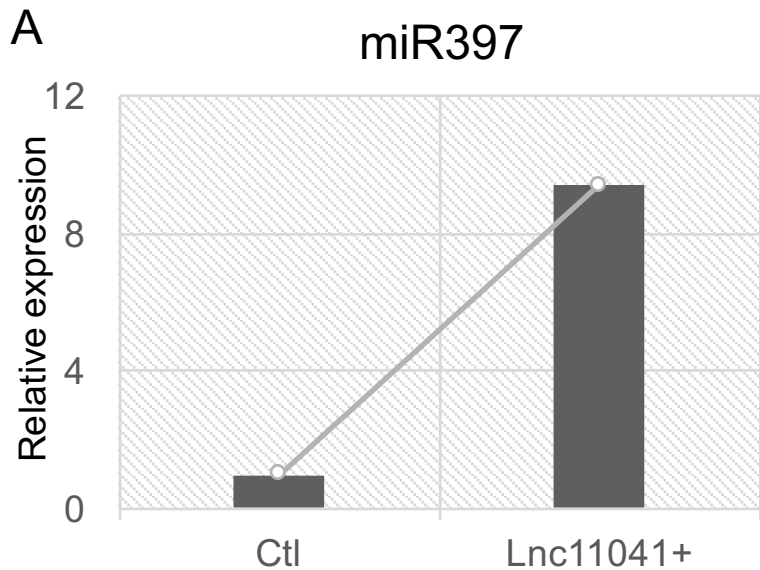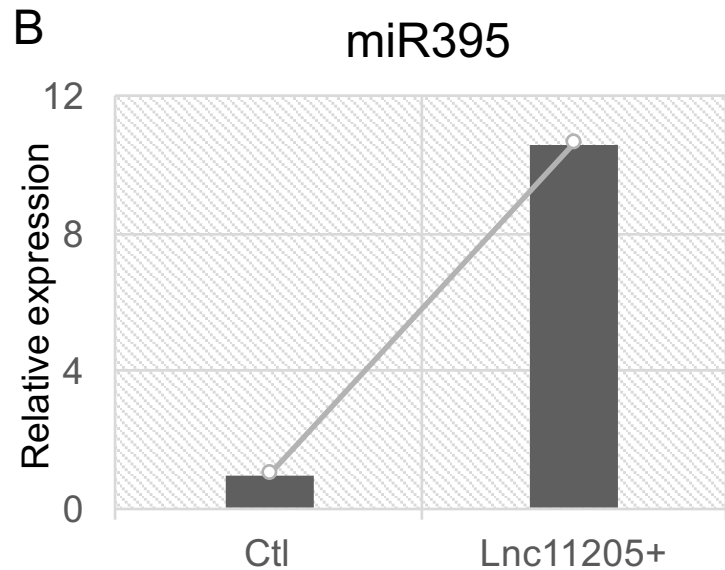

Supplement: Supplementary file 5 — Figure S4. The relative expressions of miRNAs in rubber tree mesophyll protoplasts. (A) The relative expressions of miRNA397 in rubber tree mesophyll protoplasts overexpressing lncRNA11041 (lnc11041+) and control (ctl). (B) The relative expressions of miRNA395 in rubber tree mesophyll protoplasts overexpressing lncRNA11205 (lnc11205+) and control. (PDF 12 kb) [file 13062_2019_235_MOESM5_ESM.pdf]
